# Illumination Onset: A Theoretical Neuroscience Framework for the Sudden Aha Moment

**DOI:** 10.3390/bs16101740

**Published:** 2026-09-24

**Authors:** Kyung Hee Kim

**Affiliations:** Department of School Psychology and Counselor Education, William & Mary, Williamsburg, VA 23185, USA; k@7musesinstitute.org

**Keywords:** Aha moment, creative insight, dominance inversion, edge of chaos, Illumination Onset, precision weighting, predictive processing

## Abstract

How can an Aha moment arrive after we stop trying to solve a problem? Grounded in Wallas’s account of Illumination, this theoretical neuroscience article draws on research in creativity, predictive processing, neural dynamics, and epilepsy to explain Illumination Onset, when an emerging idea is first recognized. Continued effort may keep the established understanding of a problem too dominant for alternative information to reshape it. Neuroscience studies document alpha decreases and gamma increases near reported solutions. The article terms this alpha–gamma reversal *dominance inversion* and suggests it reflects adaptive precision weighting. Top-down influence becomes less dominant but still connects the emerging idea to prior knowledge and goals. Studies link the cerebellum to prediction and correction, the anterior cingulate cortex to detecting neural surprise, and serotonin to processing it and coordinating gamma activity. The framework places Illumination Onset near the edge of chaos, where coordinated vulnerability permits thought to approach instability while remaining functionally regulated. At this threshold, the framework proposes that an allostatic whole-person transition integrates neural surprise with emotional and bodily responses and places intrinsic reward before later evaluation-related activity. Seizure onset provides a boundary comparison: Illumination remains coordinated, whereas seizure activity escapes local regulation and spreads pathologically.

## 1. Introduction

New problems and changing circumstances often require solutions that cannot be reached by applying an established answer. Information contributing to a new solution may develop gradually, yet an identifiable idea can become conscious abruptly as an Aha moment. Wallas (1926) described the final “flash,” or “click,” of illumination as “the culmination of a successful train of association” (pp. 93–94). How developing information produces this sudden conscious arrival remains unresolved.

The Neuro-Creative Cycle is a neuroscientific reinterpretation of Wallas’s creative process organized into three stages: Preparation, Imagination, and Verification (Kim, 2025a, 2025b, 2026a). Preparation establishes the knowledge, expectations, goals, and problem representation that guide creative thought. Imagination develops possible ideas through Spontaneous Imagination, Deliberate Imagination, Incubation, and Illumination, whereas Verification evaluates the resulting idea. Within Imagination, two thresholds mark successive changes in an idea’s availability. At the Creative Intimation threshold during Incubation, an idea first feels imminent but cannot yet be identified. Illumination comprises Illumination Onset, when the idea first becomes identifiable, and Illumination Experience, which follows its arrival. This article distinguishes these phases, transitions, or periods while Table 1 locates them within the complete cycle and identifies Illumination Onset as the focus of this article.

Not every sudden idea arrival is classified here as Illumination. A spontaneously arising idea becomes briefly identifiable without preceding Creative Intimation and may disappear unless recorded, as Wallas (1926) advised. By contrast, Illumination follows Creative Intimation, and the framework places its Onset within the documented response-aligned alpha–gamma time course.

Using the terminology of his time, Wallas (1926) proposed that instinctive impulses and emotions associated with the “lower brain” can initiate associations in the “upper brain,” where deliberate thought occurs (p. 32). He distinguished creative stages by their characteristic forms of thinking: Preparation depends more on deliberate thought, whereas Imagination involves greater receptivity to sensations, emotions, images, memories, and associations (Kim, 2026a). Creative thought therefore requires openness to feeling-led information. New understanding can emerge only when the established interpretation becomes sufficiently open to challenge.

This openness must remain coordinated. Too little preserves familiar assumptions and prevents innovation, whereas too much can produce ideas that lack coherence, relevance, or communicability. Wallas (1926) accordingly emphasized that intellectual independence must precede interdependence: Creators must question familiar assumptions before their ideas can be evaluated and integrated with the knowledge of others (Kim, 2025b, 2026a). He also observed that novel creative products may not be accepted until their social environments are prepared to receive them. Productive thought must therefore move beyond established interpretations while retaining enough connection with higher reasoning, prior knowledge, and shared purposes for unfamiliar information to remain meaningful. This balance between stability and openness provides the conceptual basis for locating Illumination Onset near the edge of chaos.

Allostasis translates Wallas’s whole-person coordination into neuroscientific terms. The brain anticipates changing bodily needs by coordinating perception, emotion, cognition, and action (Theriault et al., 2025). To support this coordination, the brain generates predictions, detects differences between predictions and internally or externally generated information, and determines which information should influence processing (Theriault et al., 2025). The present framework applies these processes to explain the brain’s approach to and crossing of the Illumination Onset threshold.

Within this predictive account, bottom-up information refers to embodied, emotional, imagistic, mnemonic, and associative information that contributes to a developing idea, consistent with Wallas’s (1926) emphasis on sensations and emotions during Imagination (Kim, 2025b). The term describes the direction of influence rather than the information’s external or anatomical origin: Internally generated information can function bottom-up when it challenges the prevailing interpretation. Top-down prediction refers to expectations constructed from prior knowledge that guide attention and interpretation (Katsumi et al., 2022). Within the Neuro-Creative Cycle, Preparation establishes the problem representation from which these expectations develop (Kim, 2025a, 2025b, 2026a). Prediction error is the difference between what is predicted and the internally or externally generated information that becomes available (Katsumi et al., 2022). Precision weighting determines the relative influence of predictions and prediction errors according to their estimated reliability (Lao-Rodríguez et al., 2026). A large prediction error may exert little influence if it appears unreliable, irrelevant, or incoherent, whereas a weaker signal may become influential if it consistently supports a coherent alternative relevant to the unresolved problem (Lao-Rodríguez et al., 2026).

As Illumination Onset approaches, the prevailing interpretation must become less dominant for a coherent alternative to reorganize thought. If it retains too much weight, Illumination Onset may not occur. At the Creative Intimation threshold, bottom-up information captures attention, but the prevailing interpretation remains stronger. At the Illumination Onset threshold, the framework proposes that adaptive precision weighting gives more weight to information supporting the emerging idea than to the established interpretation. Top-down influence does not disappear; it continues to help keep the idea coherent and relevant. *Dominance inversion* refers to this reversal from alpha-mediated top-down dominance to gamma-mediated bottom-up dominance. The framework locates the reversal near the edge of chaos rather than near rigid order, a regime associated with enhanced information processing (Hengen & Shew, 2025).

Allostasis provides the whole-person context for Illumination Onset. The framework proposes that idea recognition is coordinated with neural surprise arising from its departure from the prevailing interpretation, intrinsic reward, and bodily and emotional changes. It further places this Onset reward before any additional enjoyment or confidence arising from subsequent evaluation of the idea. Table 2 summarizes these principal concepts without assigning the proposed functions to any single brain region.

Contemporary insight research provides relevant evidence but has rarely isolated Illumination Onset as defined here. Insight trials are generally identified through responses made after an idea has become conscious, and neural activity is commonly aligned with those responses rather than with the idea’s arrival (Bowden & Jung-Beeman, 2007; Luo & Knoblich, 2007). Existing findings may therefore combine late Creative Intimation, Illumination Onset, subsequent confidence, and motor reporting. These studies clarify the neural activity occurring before and after insight but do not isolate the transition at which an idea becomes consciously available.

Two additional accounts clarify aspects of the proposed transition. Predictive routing explains how top-down activity regulates the feedforward transmission of unexpected information (Gabhart et al., 2025). Neural theories of consciousness distinguish subjective experience from cognitive accessibility and behavioral reporting (Storm et al., 2024). Neither account isolates the conscious arrival of a creative idea. Table 3 summarizes how the present framework integrates these accounts to explain Illumination Onset.

Figure 1 summarizes the framework across the two proposed thresholds. Panel A locates Creative Intimation, Illumination Onset, and Illumination Experience within the Neuro-Creative Cycle and shows precision weighting continuing across them. Panel B depicts the proposed reversal in relative influence: At the Illumination Onset threshold, bottom-up information overtakes the prevailing top-down prediction. This dominance inversion is proposed to occur near the edge of chaos.

Seizure onset provides a boundary comparison for distinguishing coherent neural reorganization from pathological propagation near the edge of chaos. Both transitions emerge from developing neural activity, involve some of the same neural systems and oscillatory processes, and culminate in rapid change. This does not imply that Illumination is epileptic. Section 2 examines how intrinsic engagement and passion, sensory attenuation, adaptive precision weighting and dominance inversion, cerebellar–ACC coordination, and serotonergic modulation may contribute to approaching and crossing Illumination Onset. Section 3 uses seizure onset to examine why coherent solution-related information remains coordinated at Illumination Onset, whereas pathological activity can escape local regulation and spread. Section 4 distinguishes supporting findings from new hypotheses and presents onset-centered tests. Section 5 derives implications for supporting Aha moments, and Section 6 integrates the framework into a whole-person account of sudden idea recognition.

## 2. How Does the Brain Approach and Cross the Illumination Onset Threshold?

Wallas (1926) described Illumination as the “instantaneous and unexpected” arrival of an idea and argued that this final flash could not be produced directly by an effort of will (p. 93). Preparation nevertheless remains essential because it establishes the knowledge, goals, and problem representation that make a relevant discovery possible. Conscious effort creates the conditions for an idea without determining when it will become consciously available, and the associative process can continue after deliberate analysis pauses. Existing studies have documented alpha decreases and gamma increases near reported solutions, neural reorganization before behavioral reports, greater reward activity with stronger Aha moments, and cingulate and cerebellar involvement. The subsections below treat these findings as empirical anchors and distinguish them from the framework’s proposed explanation. The framework proposes that valuing and enjoying creative activity preserve the unresolved problem’s emotional significance; alpha–beta, gamma, and theta activity organize changing informational influence; cerebellar systems help reorganize emerging idea-related information; the ACC registers neural surprise; and dorsal-raphe serotonin modifies responsiveness and gamma coordination. Their convergence at the Onset threshold may allow the emerging idea to become recognizable.

### 2.1. Intrinsic Engagement and Increasing Feeling-Led Influence

Approaching Illumination Onset may depend on both valuing and enjoying creative activity itself. Valuing the activity gives the unresolved problem personal significance and purpose; enjoying it makes exploration, imagination, and discovery intrinsically rewarding before they produce an identifiable idea (Kim, 2025a). Wallas (1914) described the highest intellectual motivation as the “passion of thought itself” rather than the pursuit of recognition or material reward (p. 246). He associated this passion with commitment to truth, delight in discovering ordered relations, and concern for the social significance of one’s work. Wallas (1921) further argued that curiosity sustains intellectual effort through pleasure and that this pleasure increases the fertility of memory and association. Enjoyment may therefore sustain the activity through which relevant information develops rather than merely follow discovery.

Passion can also influence which information contributes to an idea. Wallas (1926) proposed that sensations acquire emotional significance, emotion evokes related memories and associations, and the “drive of passion” helps direct the path of association (p. 64). After deliberate work defines the problem, emotion may preserve its aim so that associative processing remains connected to the unresolved problem without continuous focal attention. Feeling supplies motivation and personal significance, while knowledge established during Preparation supplies the concepts, goals, and problem representation that keep emerging information coherent and relevant. Wallas (1921) described creators’ best moments as those in which conscious effort joins a harmonious and enjoyable drive of the whole being. Passion therefore does more than sustain effort: It gives particular information personal value and helps feeling-led information remain connected to the unresolved problem. Passion does not replace knowledge or reasoning; it helps emotionally significant information from earlier experience contribute to reorganizing the problem representation.

An intrinsically valued problem can remain emotionally significant when it is not receiving deliberate attention (Kim, 2025a). Stepping away may reduce deliberate control while changes in bodily activity, sensation, attention, and environmental context reactivate memories, images, meanings, and associations formed through earlier work (Kim, 2026c). Wallas (1926) illustrated this process through Helmholtz’s ideas while ascending wooded hills and Poincaré’s discoveries following unsuccessful analysis and periods of military service and travel. Curiosity can motivate exploration and information seeking, while passion developed through repeated engagement can keep the unresolved problem meaningful after deliberate attention relaxes (Kim, 2026a).

General reward research helps distinguish enjoyment of an activity from pleasure obtained from its eventual outcome. Incentive-sensitization theory distinguishes mesolimbic dopamine-supported “wanting” from the hedonic “liking” experienced when a reward is consumed (Robinson & Berridge, 2025, p. 29). Actions can also acquire intrinsic motivational value when performing them is enjoyable without an explicit external outcome (Miura et al., 2017). Creative engagement may therefore continue because the activity matters to the creator and is enjoyable in itself, not only because the creator anticipates recognition, practical benefit, or pleasure after finding a solution.

Information can possess value before it resolves a problem. Participants selected options offering greater prospective information gain even when doing so required sacrificing immediate value, and these decisions engaged the ventromedial prefrontal cortex and ventral striatum (Åberg & Paz, 2024). Ventral-striatal activity increased with the expected intensity of monetary and erotic rewards signaled by predictive cues before those rewards were obtained (Sescousse et al., 2015). Curiosity enhanced incidental learning independently of monetary incentives and engaged systems supporting attention, valuation, and memory (Meliss et al., 2024). Orbitofrontal neurons also represented informational and primary-reward attributes separately during curiosity-driven decisions (Blanchard et al., 2015). Information seeking and curiosity can therefore direct motivation, attention, and memory before an outcome is available.

Value can also sustain effort. Mesolimbic dopamine release was associated with reward seeking and behavioral vigor (Mohebi et al., 2019), while dopamine systems contribute to effort-related choice (Salamone & Correa, 2024). Serotonin may contribute to value coding across the unfolding activity. In mice, dorsomedial-striatal serotonin release occurred during reward-predicting cues, approach, and consumption. The sustained magnitude of release varied with external reward value and the animal’s internal state, supporting the coding of both objective and subjective value (Spring & Nautiyal, 2024). Striatal serotonin can therefore represent value before an outcome is complete. This striatal value-related function differs from the proposed dorsal-raphe contribution to neural surprise and gamma coordination considered in Section 2.4.

Passionate engagement can also take the form of flow. Flow at work is characterized by absorption, enjoyment, and intrinsic motivation (Bakker & van Woerkom, 2017). Greater intrinsic reward, driven by a balance between task difficulty and ability, was associated with stronger functional connectivity between cognitive-control and reward systems (Huskey et al., 2018). These findings are consistent with Wallas’s (1926) account of creative activity engaging thought and feeling as an integrated whole.

Autonomic findings further suggest that creative cognition may benefit from flexible physiological regulation. Heilman (2016) proposed that creative cognition may be associated with greater parasympathetic flexibility and lower overall noradrenergic activity. In a study of divergent thinking, participants generally showed increased parasympathetic activity, while greater creative achievement predicted stronger sympathetic activation associated with more creative ideas (Silvia et al., 2014). These findings suggest that creative cognition may involve flexible autonomic adjustment rather than a uniformly relaxed or activated state.

Creativity research more directly links affective and reward processes with insight. Positive mood before problem presentation was associated with solving more problems through insight, partly through changes in anterior-cingulate activity (Subramaniam et al., 2009). Solutions producing stronger Aha experiences recruited the nucleus accumbens and ventral tegmental area more strongly than solutions producing weaker Aha experiences; caudate activity was also observed (Tik et al., 2018). Oh et al. (2020) found orbitofrontal reward activity approximately 100 ms after the principal insight signal, with stronger activity among participants with greater reward sensitivity, indicating that insight itself was more rewarding for them. The authors interpreted this timing as showing that reward was integrated with, or immediately triggered by, insight rather than produced by later conscious evaluation. They also proposed that this reward may reinforce exploration and problem solving, making future engagement more likely. This self-reinforcing function is consistent with Kim’s (2025b) interpretation of Wallas’s intellectual passion: When creative activity and discovery are valued and enjoyed in themselves, repeated intrinsic reward may sustain engagement and develop into passion.

At Illumination Onset, intrinsic reward is proposed to become integrated with idea recognition, surprise, and emotional and bodily changes before any additional enjoyment or confidence arising from subsequent evaluation.

### 2.2. Precision Weighting and Dominance Inversion

Wallas (1926) emphasized that higher forms of thought cannot always be produced directly through deliberate effort. He described Poincaré’s idea arriving as he boarded an omnibus, Helmholtz’s ideas emerging while he walked slowly, and an idea of his own arising as he began bathing. These examples illustrate idea arrival after substantial Preparation and reduced deliberate analysis.

Contemporary findings support the importance of these conditions. An undemanding task increased mind wandering and subsequent improvement on previously encountered creativity problems relative to rest, a demanding task, or no break (Baird et al., 2012). Mind wandering also predicted more creative ideas after a moderately engaging video but not after a boring video (Irving et al., 2024). Cathodal stimulation over the left prefrontal cortex facilitated uncommon tool uses, providing causal evidence that altering left-prefrontal activity can increase access to less dominant possibilities (Chrysikou et al., 2013).

Sensory attenuation provides a possible mechanism connecting familiar activity with altered information weighting. Brown et al. (2013) proposed that movement attenuates sensation by reducing the precision assigned to sensory evidence. Kiepe et al. (2021) reviewed evidence that predictable sensory input can be attenuated even when it is not produced by one’s own movement. Gu et al. (2025) identified a cerebellum-centered network involved in predicting the sensory consequences of action. Within the present framework, sensory attenuation reduces the weight of predictable sensations, allowing a slight difference—or a sensation, image, memory, or meaning associated with the solution—to become more influential. Wallas’s bathing example illustrates how familiar sensations could provide a predictable background while requiring little concentrated control.

At Creative Intimation, solution-related information is proposed to depart sufficiently from the prevailing interpretation to exceed automatic cerebellar correction, capture attention, and recruit medial-frontal processing without yet dominating thought. Precision weighting determines the relative influence of predictions and prediction errors (Lao-Rodríguez et al., 2026). Gabhart et al. (2025) proposed that top-down alpha–beta activity suppresses neuronal firing and gamma activity in pathways carrying predicted information, whereas inadequately predicted information encounters less suppression and produces stronger feedforward transmission. For internally generated images, memories, emotions, meanings, and associations, the framework proposes that alpha–beta-mediated stabilization of the prevailing interpretation weakens as gamma-mediated influence supporting the emerging idea increases.

At Illumination Onset, adaptive precision weighting completes dominance inversion by shifting dominance to gamma-mediated bottom-up information. Top-down influence remains, connecting the idea to the problem representation established through Preparation and helping preserve its coherence and relevance.

Response-aligned electroencephalography (EEG) findings provide the principal empirical basis for dominance inversion. Jung-Beeman et al. (2004) found an insight-specific posterior alpha enhancement beginning approximately 1.4 s before the solution response. This enhancement then declined and ended about 400 ms before the response, followed by a right anterior-temporal gamma burst beginning about 300 ms before it. Kounios and Beeman (2014) subsequently presented an adapted version of this time course. Figure 2 focuses on the final 500 ms, when the posterior alpha enhancement ends and the right anterior-temporal gamma burst begins, and adds an approximate threshold and shaded Illumination Onset window. The window acknowledges that response-aligned EEG cannot determine the exact moment when the idea became identifiable. Because alpha and gamma are plotted on separate vertical scales, their intersection indicates neither equal amplitudes nor an empirical threshold; it serves as a schematic marker of the proposed reversal in relative organizing influence.

Sandkühler and Bhattacharya (2008) associated restructuring with reduced right-prefrontal alpha and identified increased parieto-occipital gamma associated with correct and sudden solutions. Alpha-2 desynchronization accompanied by increased gamma-2 activity was also reported during the generation of original solutions to social problems (Pusikova & Razumnikova, 2019). These findings support the proposed shift from alpha-mediated dominance to gamma-mediated dominance, although the timing, neural locations, and experimental comparisons vary across tasks. Related findings extend but do not define dominance inversion. Sheth et al. (2009) found reduced posterior beta and increased right frontal or frontocentral gamma in several insight-relevant comparisons. Bieth et al. (2024) found a left temporocentral alpha increase followed by left-parietal gamma and then frontocentral theta before the response. Oh et al. (2020) identified prefrontal gamma approximately 500 ms before the behavioral response, and Yu et al. (2022) identified multiple oscillatory states across problem solving.

Theta may organize emerging information without directly producing dominance inversion. Theta–gamma coupling has been proposed as a mechanism for organizing information sequences and updating predictive models (Köster, 2024), and cross-regional theta–gamma coupling has been observed in error monitoring (Moser et al., 2024). Extending these findings, the framework proposes that frontal theta supports the integration of idea-related information as gamma-mediated influence increases near Illumination Onset.

Brain-stimulation findings provide causal evidence that temporal-cortical activity contributes to some forms of insight. Gamma-frequency stimulation over the right temporal cortex increased verbal-insight solutions and bilateral temporal gamma activity (Santarnecchi et al., 2019). Cathodal stimulation of the left anterior temporal lobe combined with anodal stimulation of the right anterior temporal lobe also facilitated matchstick and nine-dot insight solutions (Chi & Snyder, 2011, 2012).

Studies tracking neural patterns provide complementary evidence of rapid reorganization. Stronger Aha moments were associated with greater pre- to post-solution representational change, network integration, hippocampal and amygdala activity, and subsequent memory (Becker et al., 2025). During narrative comprehension, reinstatement of causally relevant earlier information preceded a rapid neural change approximately two seconds before an Aha response (Song et al., 2026). Together, these findings show that reorganization can precede behavioral reporting, but they do not identify Illumination Onset or demonstrate dominance inversion.

The framework proposes a change in relative hemispheric influence rather than exclusive control by either hemisphere. Aziz-Zadeh et al. (2009) found early left-insular activity for search solutions, and Bieth et al. (2024) found left temporocentral alpha before reported insight. The framework interprets these findings as consistent with a left-hemisphere contribution to preserving the prevailing problem representation before the behavioral response, although they do not establish whether this activity precedes Illumination Onset. Right anterior-temporal gamma and broader activation of remotely related meanings support a task-dependent right-hemisphere advantage in organizing previously subordinate relations, particularly in verbal insight, while left-sided pre-response gamma shows that this advantage is not exclusive (Bieth et al., 2024; Bowden et al., 2005; Jung-Beeman et al., 2004; Kounios & Beeman, 2014). Sprugnoli et al. (2017) documented distributed bilateral activity across insight studies. Because those studies generally aligned neural activity with solution reports, these findings may include both the hemispheric reorganization proposed at Illumination Onset and the broader bilateral integration proposed for Illumination Experience.

### 2.3. Proposed Cerebellar–ACC Coordination at Illumination Onset

Creativity research implicates the cerebellum in creative thinking. Stronger figural-creativity performance was associated with more efficient communication across networks that included the cerebellum (Gao et al., 2017). Creative-capacity training increased cerebellar–cerebral connectivity during spontaneous figural improvisation (Saggar et al., 2017). Divergent-thinking performance was associated with Crus I–II network connectedness, with stronger Crus I connectivity to cerebral association regions among higher-performing participants (Gao et al., 2020). Better insightful-problem-solving performance was associated with lower resting-state connectedness in a network encompassing the brainstem, cerebellum, and thalamus, indicating that performance depends on how this network is organized rather than simply on greater connectivity (Lin et al., 2022).

Building on Wallas’s (1926) whole-person account of Illumination, the present framework examines the cerebellum’s contribution to distributed coordination at Illumination Onset. The cerebellum is part of a distributed whole-brain system that supports allostasis (Theriault et al., 2025). Katsumi et al. (2022) further proposed that cerebellar functional organization may contribute to constructing and modifying predictions, prediction errors, and precision signals in service of allostasis. These functions provide a neuroscientific basis for the proposed contribution of cerebellar–ACC coordination to Illumination Onset.

A memory-search study provides more temporally specific evidence. Lobule VI activity increased as participants approached a change in search, providing a closer parallel to Creative Intimation. Crus I–II activity also increased before some changes but was most clearly associated with the transition from the existing search pattern to a new one (Lundin et al., 2023). The pattern reported by Lundin et al. (2023) makes Crus I–II particularly relevant to the framework’s account of Illumination Onset, when emerging idea-related information becomes the stronger organizer of thought. Crus I–II occupy nonmotor cerebellar territories connected with higher-order association networks (Guell et al., 2018). In patients with cerebellar degeneration, reduced Crus I–II gray-matter volume was associated with weaker feedback-related negativity—a neural response to differences between expected and actual outcomes linked to medial-frontal and ACC processing (Berlijn et al., 2025).

The ventral dentate nucleus (DN) provides a major output route from cognitive cerebellar regions and belongs to a functional network that includes Crus II and the ACC (Kulkarni et al., 2023). This network provides evidence that cognitive cerebellar regions and the ACC participate in a shared functional system. Ventral-DN lesions impaired working memory and the ability to change rules or strategies, increased repeated use of ineffective responses, and relatively spared fine motor control and recognition memory (Schmahmann et al., 2025). During prism adaptation, the ventrocaudal DN was recruited early as participants made rapid strategic corrections to altered visual feedback (Küper et al., 2014). Experimental manipulation of cerebellar output altered theta- and gamma-band coherence between cortical regions (McAfee et al., 2022). Cerebellar damage and temporary disruption altered ACC-associated error processing (Huvermann et al., 2025). Cerebellar damage also altered the relationship between medial-prefrontal theta activity and performance during error monitoring (Andre et al., 2024). These findings provide possible routes through which cerebellar systems may influence cortical coordination and ACC-related processing as an established organization changes.

Andre et al. (2024) provide a domain-general precedent for threshold-dependent recruitment. Frontal-midline theta emerged when prediction error exceeded the capacity of automatic cerebellar correction, indicating recruitment of medial-frontal processing. Applied to the present framework, routine cerebellar prediction and correction may continue as a solution develops. At Creative Intimation, the discrepancy becomes sufficient to exceed automatic correction and recruit medial-frontal processing, including the ACC, although the prevailing interpretation remains dominant. At Illumination Onset, adaptive precision weighting makes the solution-related information dominant and is proposed to produce a stronger ACC-related surprise response. Insight findings provide complementary support for this proposal. Medial-frontal activity was associated with internally driven strategy shifts and representational change during insight problem solving (Shen et al., 2018). ACC activity increased when the structure underlying an insightful solution had not been anticipated, providing an insight-specific precedent for the proposed ACC response to surprise (Luo et al., 2004).

Insight-producing riddle answers elicited an ACC response associated with breaking a mental set (Mai et al., 2004). Anagrams solved through insight recruited the ACC more strongly than those solved through deliberate search (Aziz-Zadeh et al., 2009). A meta-analysis of functional magnetic resonance imaging (fMRI) studies of insight identified convergent activity in the cingulate cortex and cerebellum (Sprugnoli et al., 2017). The proposed ACC function differs across the two thresholds. During Creative Intimation, the ACC may maintain attention to emerging information that conflicts with the prevailing interpretation. At Illumination Onset, it may respond to the resulting surprise and support the shift to the new idea. Vassena et al. (2020) found that surprise best explained dorsal-ACC activity during rapid decisions requiring cognitive control. In mice, larger ACC prediction-error signals preceded successful one-trial rule changes and declined after the new rule was adopted, supporting a brief role in transition rather than maintenance (Cole et al., 2024). A computational model proposes that the ACC tracks Bayesian surprise—the extent to which new information changes an existing expectation—and adjusts cognitive control accordingly (Vriens et al., 2025).

Based on these findings, the framework proposes that Crus I–II and ventral-DN output help organize a coherent alternative and coordinate it with cerebral association systems. The ACC may register how substantially the alternative changes the prevailing expectation and promote the abrupt shift away from the established interpretation. Within the distributed allostatic system, this cerebellar–ACC coordination may help align representational reorganization with broader cognitive, affective, and bodily regulation. Their coordination may allow the emerging idea to organize thought and become consciously identifiable.

### 2.4. Serotonergic Modulation of Surprise and Gamma Coordination

The serotonergic contribution proposed for Illumination Onset concerns the dorsal raphe, which may alter how the brain responds to unexpected information and coordinates the resulting activity. This differs from dorsomedial-striatal serotonin associated with reward-predicting cues, approach, consumption, and subjective value (Spring & Nautiyal, 2024), discussed in Section 2.1 in relation to intrinsic engagement.

Most serotonin-producing neurons are concentrated in the brainstem raphe nuclei. The dorsal raphe projects widely across the brain, allowing serotonin to modify cortical, limbic, sensory, and subcortical activity. Its effects depend on the receptor, cell type, region, and current network state (Celada et al., 2013). Animal studies identify several dorsal-raphe functions that may be relevant near Illumination Onset. Serotonergic neurons responded when outcomes differed from expectations and supported adaptation when learned relationships changed (Matias et al., 2017). Their activity also tracked uncertainty and influenced how rapidly behavior was updated (Grossman et al., 2022). Computational modeling indicated that dorsal-raphe serotonin-neuron activity represents the prospective value of near-future outcomes (Harkin et al., 2025). Within the framework, this value signal may help prioritize an emerging idea as its significance increases.

Serotonin can also affect which information receives attention. Rapid suppression of dorsal-raphe serotonergic activity predicted better visual attention in mice, and experimental suppression improved performance by reducing competition among sensory signals (Lehnert et al., 2026). Conversely, stimulating dorsal-raphe serotonergic neurons increased activity across widespread cortical and subcortical regions in awake mice (Hamada et al., 2024). Dorsal-raphe serotonin may therefore modify both information selection and the responsiveness of distributed neural systems.

Many cortical and limbic systems modulated by raphe serotonin are also recruited during insight (Sprugnoli et al., 2017). Insight studies have additionally reported activity in the midline pons (Aziz-Zadeh et al., 2009) and red nucleus (Luo et al., 2004). Brainstem–cerebellar–thalamic network organization has also been associated with insight-problem-solving performance (Lin et al., 2022). This anatomical overlap supports investigating whether serotonin modulates distributed insight-related activity.

Illumination Onset is not equated with a psychedelic state; psychedelic findings are used here only to clarify possible serotonergic functions. Psychedelic research illustrates how strong serotonergic-receptor activation can shift the balance between bottom-up information and top-down prediction. Serotonergic psychedelics such as psilocybin and LSD produce their characteristic effects largely by activating 5-HT2A receptors (De Filippo & Schmitz, 2024). Predictive accounts propose that this activation weakens the constraint imposed by established high-level predictions, allowing sensory information and prediction errors to exert greater bottom-up influence (Carhart-Harris & Friston, 2019). Psilocybin altered tactile prediction-error processing and the integration of sensory input with prior beliefs, providing experimental evidence that 5-HT2A activation changes this balance (Duerler et al., 2022).

De Filippo and Schmitz (2024) describe *synthetic surprise*, in which serotonergic alteration makes sensory or internally generated activity differ from what the brain predicts even without an unexpected external event. Because the brain treats this activity like unexpected evidence, it can influence the established prediction. Serotonergic psychedelics also altered large-scale network integration and segregation (Luppi et al., 2021). They disrupted individuals’ established patterns of functional connectivity (Siegel et al., 2024). Their effects can weaken some predictions while strengthening others depending on dose, context, neural system, and hierarchical level (Safron et al., 2025). Applied to Illumination Onset, the framework proposes that a subtler endogenous serotonergic process may allow internally generated information to acquire unexpected significance and greater bottom-up influence. Synthetic surprise provides a possible model of how an idea can challenge the prevailing interpretation despite the absence of an unexpected external event.

Section 2.2 proposed that gamma activity helps emerging information gain feedforward influence. Serotonin may affect how this gamma activity is timed and synchronized. Gamma oscillations depend partly on precisely timed interactions between excitatory pyramidal neurons and inhibitory fast-spiking interneurons (Buzsáki & Wang, 2012). In the mouse prefrontal cortex, 5-HT2A-receptor activation enhanced fast-spiking interneurons’ responses to gamma-frequency input, demonstrating one way serotonergic activity can modify gamma timing (Athilingam et al., 2017).

Psychedelic studies show that serotonin-related effects on gamma involve organization as well as power. Psilocybin reduced the trial-to-trial timing consistency of human 40 Hz auditory responses without significantly changing their power (Viktorin et al., 2022). Psilocin similarly reduced 40 Hz timing consistency in the right temporal cortex of rats without consistently changing power (Griškova-Bulanova et al., 2026). In contrast, DMT increased spontaneous gamma power while reducing alpha power and increasing neural-signal diversity (Timmermann et al., 2023). Serotonin may therefore influence the timing and synchronization of gamma activity, not merely its strength. Within the framework, precisely coordinated gamma may help transmit a coherent emerging idea, whereas greater gamma power alone does not ensure coherent information transfer.

The framework proposes two complementary serotonergic contributions near Illumination Onset. Prospective-value coding may help prioritize an emerging idea, while surprise processing may help it challenge the prevailing prediction. Serotonin may also modify the gamma coordination through which the brain gives more weight to this information. It does not generate the idea or constitute dominance inversion. Figure 3A depicts serotonergic modulation of cellular interactions supporting local gamma, while Figure 3B depicts the proposed changes in serotonin, theta, alpha, and gamma around the Onset threshold.

## 3. How Do Illumination Onset and Seizure Onset Diverge near the Edge of Chaos?

Seizure onset provides a boundary comparison for clarifying why susceptibility to neural reorganization can culminate in either a functional or pathological transition. Both Illumination Onset and seizure onset emerge from developing neural activity, culminate in a rapid change, and may involve partly overlapping cerebellar–ACC, oscillatory, and serotonergic processes. Some focal seizures are consciously experienced from their onset as an aura, even before wider propagation or visible motor signs (Fisher, 2017).

Wallas’s (1926) account provides the conceptual basis for this distinction. Openness allows unfamiliar information to challenge an established interpretation, but productive thought remains guided by the creator’s broader knowledge and purposes. The present framework describes this productive openness as coordinated vulnerability: increasing susceptibility to reorganization while retaining the constraints needed for emerging information to remain coherent and relevant. As Illumination Onset approaches, the problem representation established during Preparation becomes progressively more open to a coherent alternative. This state is proposed to occur near the edge of chaos, where susceptibility to change increases without losing functional integration.

Research on metastability and criticality helps explain how neural systems can become more responsive while preserving functional organization. Metastability allows the brain to move among temporary but coordinated patterns of neural activity (O’Byrne & Jerbi, 2022). Near a critical transition, small inputs can produce comparatively large changes, increasing sensitivity and dynamic range (Kinouchi & Copelli, 2006). Neural avalanches—cascades of activity across connected neural populations—illustrate how activity can propagate near criticality (Plenz et al., 2021). Information richness may also increase near the boundary between excessive regularity and excessive instability (Toker et al., 2022).

Computational neuron models provide additional evidence that responsiveness depends on activity remaining within a functional range. Presynaptic activity at an intermediate level improved weak-signal detection in one model (Baysal, 2024). In another, chaotic background spike trains enhanced signal processing when activity remained within an appropriate range (Baysal et al., 2026). These intermediate activity levels do not place Illumination Onset midway between order and instability; the framework locates it close to instability while coordinated processing is preserved. The models provide computational precedents for state-dependent sensitivity to previously weak information.

Greater sensitivity does not determine which information gains influence or whether the resulting transition remains coordinated. Precision weighting determines the relative influence of information according to its estimated reliability (Lao-Rodríguez et al., 2026). At Illumination Onset, the framework proposes that coherent, problem-relevant bottom-up information becomes more influential than the prevailing top-down prediction, while top-down influence remains active and connects the emerging idea with prior knowledge and current goals. At seizure onset, pathological activity may instead gain disproportionate influence and recruit additional neural populations.

Seizure onset is not simply a movement toward ever-greater chaos. Dynamical models describe it as a transition from non-seizure activity into a distinct seizure state (Jirsa et al., 2014), and an analysis of more than 2000 focal seizures identified both gradual and abrupt transitions (Saggio et al., 2020). After generalized seizures began, neural dynamics shifted away from an information-rich, near-critical regime and became more regular and repetitive (Toker et al., 2022). These findings establish that seizure onset is a dynamical transition and that post-onset activity can move away from a near-critical regime. The present framework further proposes that susceptibility near a critical boundary can culminate either in coherent idea arrival at Illumination Onset or in increasingly synchronized, repetitive, and information-poor activity following seizure onset. Table 4 summarizes the shared features of these transitions and the conditions proposed to distinguish their outcomes.

### 3.1. Cerebellar–ACC Contributions to Divergent Transitions

Cerebellar abnormalities occur across several forms of epilepsy. Kerestes et al. (2024) found especially pronounced volume loss in posterior cerebellar territories, including bilateral Crus I–II; greater loss was associated with younger age at first seizure and longer illness duration. Ibdali et al. (2021) similarly identified cerebellar degeneration across the epilepsy literature. Allen et al. (2019) reported substantial cerebellar volume loss in people who died from sudden unexpected death in epilepsy, with less pronounced differences in a group considered at elevated risk.

Experimental studies show that cerebellar output can influence seizure initiation and suppression. In a mouse model, silencing cerebellar nuclei blocked the initiation of experimentally elicited generalized convulsive seizures (Beckinghausen et al., 2023/2024). In a mouse model of temporal-lobe epilepsy, stimulating fastigial neurons projecting to the central lateral thalamus suppressed hippocampal seizures, whereas stimulating pathways to the superior colliculus or medullary reticular formation did not (Streng et al., 2021).

Human findings further implicate distributed cerebellar and cingulate relationships. Dos Santos Silva et al. (2024) identified gray-matter abnormalities across regions that included the cerebellum, cingulate gyrus, parahippocampal gyrus, thalamus, and striatum in mesial temporal-lobe epilepsy with hippocampal sclerosis. Reduced connectivity between right Crus II and the left parahippocampal cortex was associated with measures of attention and executive function, visuospatial ability, and working memory (Wang et al., 2023). Weaker connections between the right DN and frontal, temporal, and posterior-cingulate regions distinguished people whose focal seizures developed into bilateral tonic–clonic seizures from those whose seizures remained focal (Nie et al., 2022). Seizures arising from the ACC can also begin with emotional and interoceptive auras (Pana & Nguyen, 2023).

Building on these findings, the framework proposes that the organization and regulation of partly overlapping cerebellar–ACC systems distinguish coherent idea-related reorganization and neural surprise at Illumination Onset from pathological network activity at seizure onset. This proposed distinction requires direct comparative testing.

### 3.2. Oscillatory Organization and Serotonergic Susceptibility

Alpha–beta activity may either restrict abnormal influence or become incorporated into seizure activity. Pellegrino et al. (2021) observed brief posterior alpha–beta responses after frontal discharges occurring between seizures. Larger responses were associated with stronger cognitive performance, consistent with reduced disruption outside the originating region. Experimentally generated cortical seizures included 10–15 Hz fast runs originating within the neocortex (Timofeev et al., 1998). Inhibitory periods during these seizures became too brief to suppress recurrent fast activity, allowing seizure activity to continue (Timofeev et al., 1998). Beta-frequency activity was also common at frontal-lobe seizure onset, and onset patterns varied with frontal location (García-López et al., 2021).

Epilepsy studies also report gamma-range activity. Hughes (2008) reviewed gamma activity between 25 and 100 Hz in relation to epileptiform activity. Lévesque et al. (2025) described low-voltage fast activity in the 30–100 Hz beta–gamma range as a common focal-seizure-onset pattern. Worrell et al. (2004) localized 60–100 Hz high-frequency epileptiform activity to neocortical seizure-onset tissue and found that it increased before many seizures. Ren et al. (2015) found that 30–100 Hz gamma oscillations preceding some interictal discharges were strongly associated with the seizure-onset zone.

Epileptic activity also occurs above the gamma range. Jirsch et al. (2006) detected oscillations between 100 and 500 Hz near focal-seizure onset, and high-frequency oscillations (HFOs) can emerge before or around temporal-lobe seizures (Mokhothu & Tanaka, 2021). Physiological and epileptic HFOs overlap in frequency, although fast ripples are more concentrated in epileptogenic tissue (Zijlmans et al., 2012). Frequency alone therefore cannot distinguish coordinated communication from pathological synchronization.

Serotonergic state provides another source of variation in neural susceptibility. In a kindling model, repeated hippocampal stimulation produced progressively greater seizure susceptibility together with increased activity among dorsal-raphe serotonergic neurons. Suppressing those neurons reduced both seizure development and severity (Cheng et al., 2021). Serotonergic effects on seizures vary with the receptors, regions, seizure models, and experimental conditions involved (Bagdy et al., 2007; Sourbron & Lagae, 2022).

The framework proposes that, near Illumination Onset, alpha–beta-mediated top-down influence decreases as gamma-mediated bottom-up information becomes dominant, leading the brain to give more weight to coherent idea-related information. At seizure onset, gamma and faster activity may instead become organized within a pathological network and recruit additional regions. The functional outcome therefore depends on what information the activity carries, how that information is weighted, and how the activity is regulated and propagated. Whether serotonin contributes to this proposed divergence remains untested.

### 3.3. Selection of Information Versus Pathological Spread

In a study of interictal focal-epilepsy networks, Omidvarnia et al. (2017) proposed that excessive precision assigned to local prediction-error signals may strengthen local connectivity while weakening communication across the cortical hierarchy. The present comparison extends this account by hypothesizing that, near seizure onset, excessive weighting of these signals could allow pathological bottom-up activity to dominate as broader top-down constraints weaken.

Local neural excitation–inhibition represents another dimension of seizure susceptibility. Here, excitation–inhibition refers to activity within epileptogenic circuitry; it differs from the relative top-down–bottom-up influence proposed for Illumination Onset. Cortical-interneuron dysfunction has been linked to altered excitation–inhibition balance in epilepsy associated with autism spectrum disorder (Jacob, 2016). In an intracranial EEG study of mesial temporal-lobe epilepsy, measures intended to index local inhibitory and excitatory activity helped identify the epileptogenic zone (Nagata et al., 2024). Cross-regional synchronization may mark propagation beyond local circuitry. Misra et al. (2018) found that coupling between a neocortical seizure focus and mesial-temporal regions increased before those downstream regions became involved.

Pathological spread can interfere with the information ordinarily processed by recruited regions. Raghavan et al. (2024) found that interictal gamma amplitude-envelope correlations were markedly elevated among recording sites within seizure-onset zones. Epileptic HFOs impaired hippocampal information coding in rats with chronic epilepsy (Ewell et al., 2019). In humans, spontaneous epileptic HFOs temporarily delayed or prevented expected neural responses during cognitive processing (Liu & Parvizi, 2019). Das and Luczak (2022) proposed that epileptogenesis may hijack physiological memory-consolidation processes to strengthen epileptic circuits.

Epilepsy research directly establishes that pathological activity can become synchronized, recruit downstream regions, and disrupt the information those regions ordinarily process. Building on that evidence, the framework hypothesizes that the two transitions diverge through differences in precision weighting and propagation regulation. At Illumination Onset, the brain is proposed to give more weight to coherent, problem-relevant information while prior knowledge and current goals continue to constrain the emerging idea. At seizure onset, excessive precision may instead amplify pathological activity, allowing it to escape local regulation and recruit additional regions. Direct comparative tests must determine whether this proposed difference in information selection, weighting, and propagation explains the divergent outcomes.

## 4. Empirical Support, Limitations, and Tests of the Illumination Onset Framework

Section 2 and Section 3 reviewed evidence from studies that measured brain activity around the time participants reported a solution, as well as from animal studies, psychedelic research, epilepsy research, and tasks not involving creativity. These findings support individual components of the framework in the contexts in which they were studied but do not establish their proposed coordination at human Illumination Onset. The central empirical task is therefore to identify an Onset window independently of response timing and test whether the components converge as proposed. Table 5 specifies the findings that would support or challenge each claim.

### 4.1. Identifying Illumination Onset

An Onset window cannot be inferred directly from a button press or spoken answer because the response follows idea recognition and may include response preparation or early Illumination Experience. Preregistered criteria should combine subjective reports with neural change points and response-preparation markers. Immediate, delayed, and end-of-trial reporting conditions can determine whether reporting alters the measured process.

Reports should distinguish feeling that an idea is approaching, recognizing the idea, subjective surprise and pleasure, emotional and bodily changes, and later confidence and evaluation. Analytic solutions, incorrect Aha responses, non-illumination trials, and spontaneous idea arrivals can determine whether candidate markers are specific to Illumination Onset rather than reflecting recognition, suddenness, confidence, or movement more generally.

### 4.2. Testing the Proposed Mechanisms

Oscillatory power alone cannot establish the direction of information flow. Tests of dominance inversion should therefore combine artifact-controlled EEG or MEG with neural decoding and measures of directed connectivity while manipulating the strength of the prevailing interpretation and the reliability of emerging information. Decoding should determine whether the original problem representation remains detectable, while independent ratings should determine whether the resulting idea satisfies the problem’s constraints. Cross-regional analyses should test whether theta tracks coordination rather than dominance inversion itself. Scalp-recorded gamma can be contaminated by cranial-muscle activity; muscle, eye-movement, and other artifacts must therefore be controlled before gamma changes are interpreted as neural activity (Muthukumaraswamy, 2013).

Temporal order would support the proposed progression but would not establish causation. Controlled perturbation should follow the identification of reliable temporal associations. Verbal and nonverbal tasks are also needed to determine whether the proposed hemispheric progression is task-dependent.

Sensory attenuation must be demonstrated through reduced neural responses to predictable sensations before testing whether it selectively increases the relative influence of solution-related rather than unrelated information and facilitates solution recognition.

Regional coactivation cannot establish the proposed cerebellar–medial-frontal–ACC sequence or demonstrate a causal contribution to Onset. Temporally resolved multimodal measurements are needed to distinguish the proposed processes, followed by controlled perturbation to test causation.

Recognition and evaluation should be separated experimentally so that the beginning of evaluation can be compared with reward, serotonin, neural surprise, and autonomic changes. After accounting for measurement latency, components that consistently begin only after idea recognition should be assigned to Illumination Experience rather than Illumination Onset.

### 4.3. Distinguishing Illumination Onset from Seizure Onset

The proposed near-critical location of Illumination Onset requires converging evidence across multiple dynamical measures rather than reliance on a single indicator. Criteria for the relevant range should be preregistered and should distinguish heightened susceptibility with preserved coordination from rigid or disorganized activity.

Comparisons with seizure onset require matched temporal windows and clear separation of electrographic onset, consciously experienced aura, observable clinical onset, and subsequent propagation. Overlapping frequency ranges cannot establish a shared function; the comparison must instead examine how information is weighted, coordinated, and propagated.

## 5. Implications for Supporting Aha Moments

Supporting Illumination Onset begins with genuine personal interest in the problem or creative activity. That interest provides the basis for intrinsic engagement, directs curiosity toward unresolved questions and unfamiliar information, and allows passion to keep the problem meaningful when deliberate analysis stops. Sufficient Preparation provides the knowledge, skills, problem definition, and goals needed for a relevant idea to develop. Once this foundation is adequate, time away from deliberate analysis allows associative processing to continue. If it is inadequate, further Preparation is needed.

When deliberate analysis repeatedly returns to the same interpretation, people may benefit from stopping the effort to force a solution. Open-monitoring meditation distributes attention across thoughts, sensations, and feelings without making any one the primary focus and may reduce automatic habits of thought and increase thought spontaneity (Christoff Hadjiilieva, 2025). Within the present framework, it may therefore help prevent habitual thinking from preserving the prevailing interpretation during Imagination. Tolerating uncertainty and becoming vulnerable to the possibility that familiar beliefs and expectations may be wrong can help sustain this loosening. Openness without immediate judgment lets sensations, emotions, images, memories, meanings, and associations enter attention before people decide their relevance. Creative engagement may help regulate difficult emotions and bodily arousal (Kim, 2026b). These functions may allow receptivity to remain coordinated as uncertainty increases.

Meaningful variation may further loosen the prevailing interpretation. Changing perspective, representational form, bodily activity, or surroundings can make different information available without requiring continued analysis. Repeating the same strategy may preserve the impasse, but unlimited variation may produce distraction or incoherence. The practical aim is coordinated vulnerability near the edge of chaos: close to instability while the coherence provided by Preparation is preserved. If thought is already scattered or incoherent, the person may need to reduce stimulation or return attention to the problem’s constraints.

A familiar, moderately engaging activity, such as walking or bathing, may occupy enough attention to limit unrelated worries while keeping deliberate analysis relaxed. By giving predictable sensations less weight, sensory attenuation may make solution-related information easier to notice. People should not monitor continuously for an Aha moment because doing so may reinstate deliberate control.

When working alone, people can move among words, images, objects, movements, analogies, or spatial representations rather than mechanically repeating one analytic strategy. When other people are present, anticipated criticism, pressure to explain, or demands for immediate originality may reactivate self-monitoring and self-censorship. Privacy or a psychologically safe environment may allow unfinished ideas to remain unexplained until the creator is ready. People should retain control over whether and when an incomplete idea is shared; vulnerability must remain voluntary rather than become another performance demand.

Support for Illumination Onset should not be limited to researchers, inventors, entrepreneurs, artists, business professionals, and other occupational problem solvers. It should also extend to older adults and patients. Changes in movement, sensation, energy, attention, or memory may alter which activities preserve coordinated receptivity. Kim (2026b) reviewed evidence that creative activities can support attention, memory, movement, sleep, and restoration in later life. These benefits may strengthen Preparation and bodily readiness for Imagination. Kim (2025a) recommended tailoring creative activities to the interests, abilities, and emerging strengths of people with Parkinson’s disease. Families, caregivers, clinicians, educators, and community programs can preserve agency and intrinsic engagement by providing adapted materials, manageable activities, and meaningful choices rather than imposing a fixed method or performance standard.

When an Aha moment occurs, the person can notice the surprise and emotional and bodily responses and enjoy the intrinsic reward without immediately evaluating the idea. After the initial response has unfolded, Verification remains necessary to test, refine, and communicate it.

## 6. Conclusions

Building on Wallas’s (1926) account of Illumination as the sudden arrival of an idea that cannot be produced directly through deliberate effort, the present framework identifies Illumination Onset as the moment when developing information first becomes identifiable as an idea. Illumination Onset follows Creative Intimation, when an idea feels imminent but remains unidentified, and precedes Illumination Experience.

Documented alpha and gamma findings provide the empirical basis for the framework’s dominance-inversion account of Illumination Onset—the shift from alpha-mediated top-down dominance to gamma-mediated bottom-up dominance. The framework proposes that this shift reflects adaptive precision weighting. Alpha–beta activity helps stabilize the prevailing interpretation, gamma-mediated activity organizes and transmits information supporting the emerging idea, and theta may coordinate this information across distributed regions. Top-down influence becomes less dominant but does not disappear. As deliberate analysis relaxes, sensory attenuation may reduce the weight of predictable sensations and make weak solution-related information more noticeable.

The framework further proposes that cerebellar systems support prediction and automatic correction. When solution-related information persists beyond this correction, it captures attention and recruits ACC-related processing at Creative Intimation. At Illumination Onset, the framework proposes a stronger ACC-related neural-surprise response and distinguishes value-related striatal serotonin from dorsal-raphe modulation of unexpected information and gamma organization. Within an allostatic whole-person account, these processes are proposed to coordinate idea recognition with intrinsic reward and emotional and bodily responses before later evaluation-related neural activity.

Illumination Onset is located near the edge of chaos—close to instability while functional coordination remains preserved. Intrinsic engagement and passion keep the unresolved problem meaningful when deliberate attention shifts elsewhere, while tolerance of uncertainty and Openness permit vulnerability to substantial reorganization. Preparation keeps the emerging idea coherent and relevant. This coordinated vulnerability distinguishes Illumination Onset from seizure onset, in which pathological activity escapes local regulation and spreads. Together, these elements explain how prior development can culminate in an Aha moment.

## Figures and Tables

**Figure 1:**
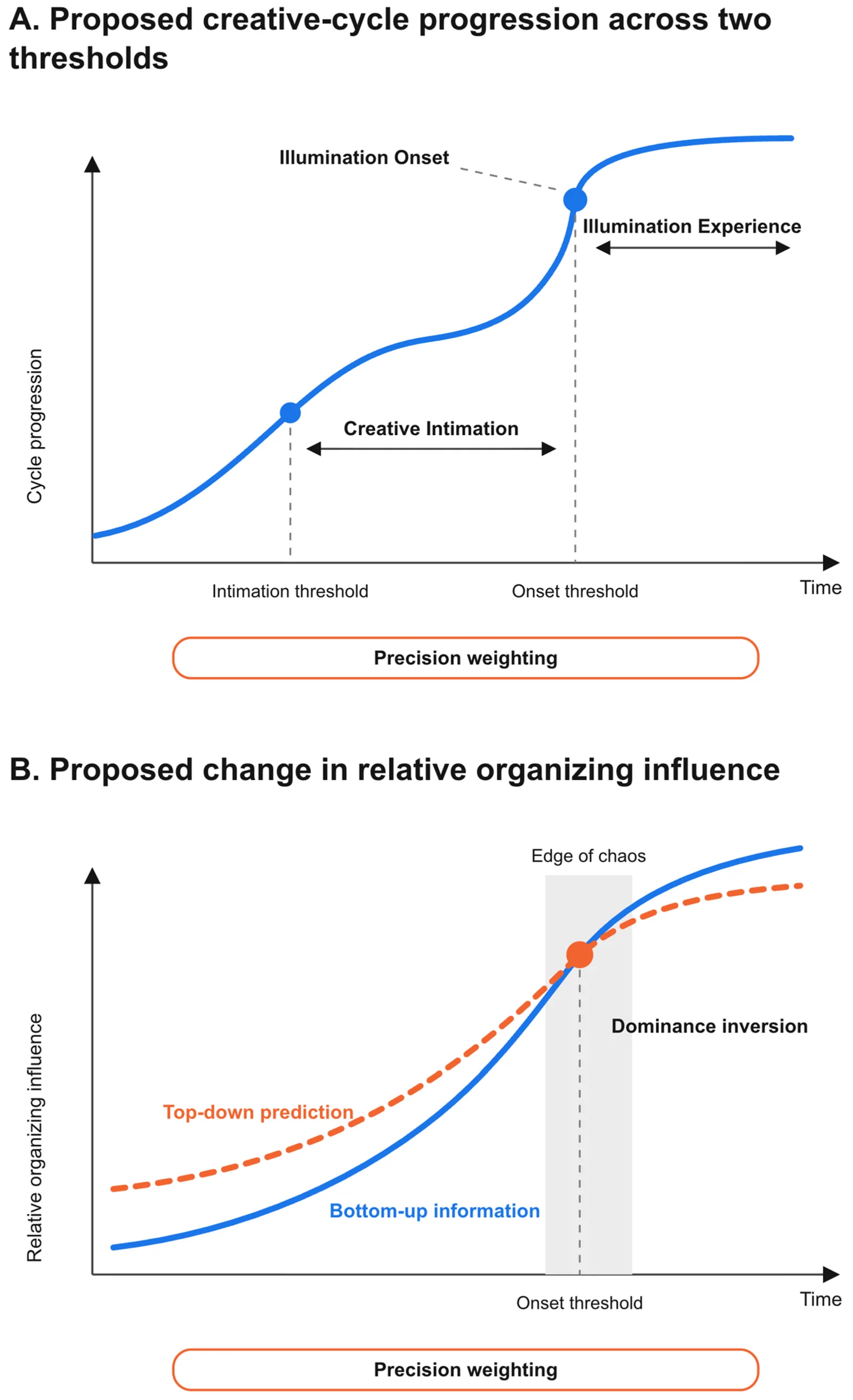
Proposed progression across two thresholds and precision weighting at Illumination Onset.

**Figure 2:**
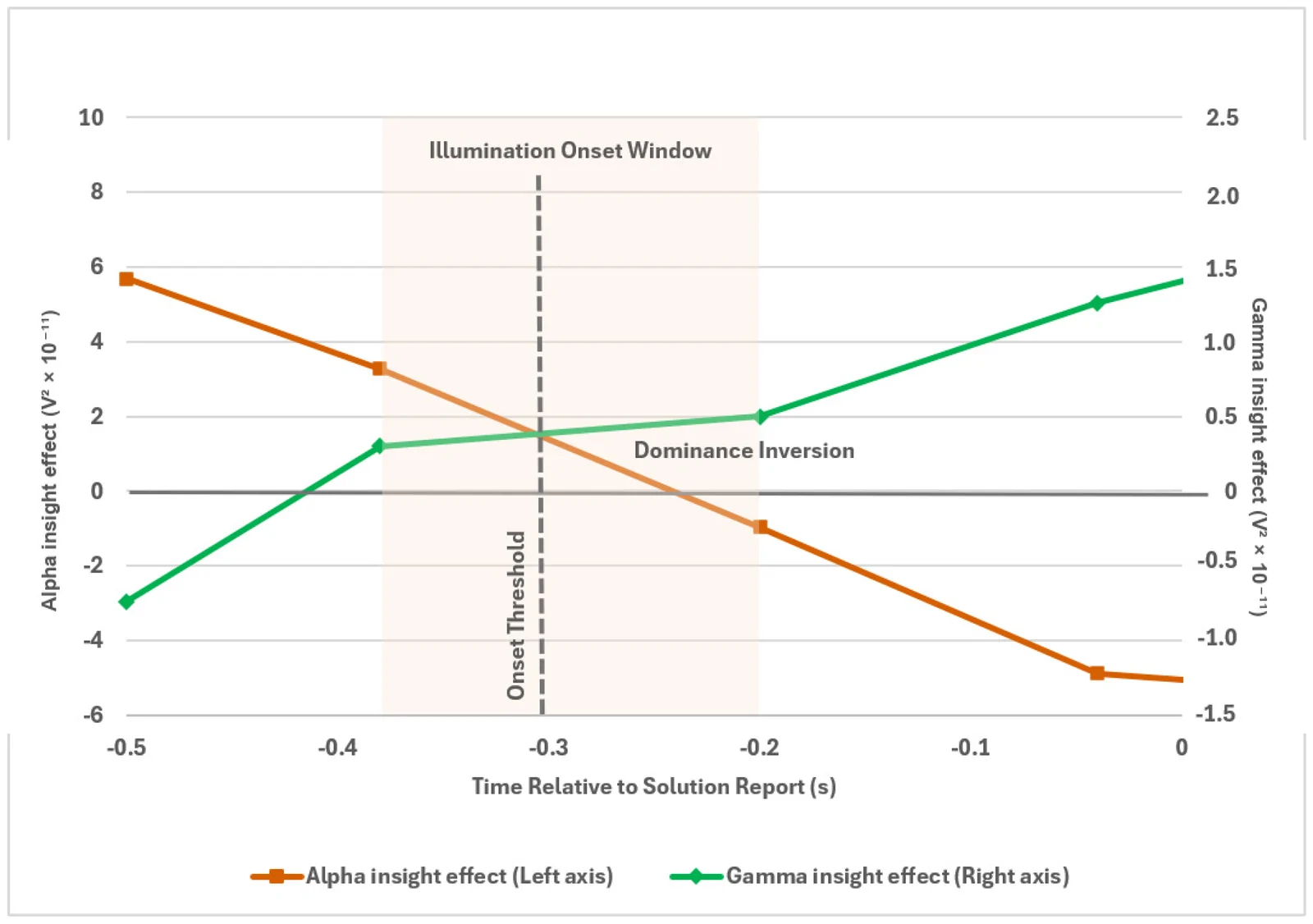
Empirical alpha–gamma time course and its proposed relation to Illumination Onset informed by Jung-Beeman et al. (2004) and Kounios and Beeman (2014).

**Figure 3:**
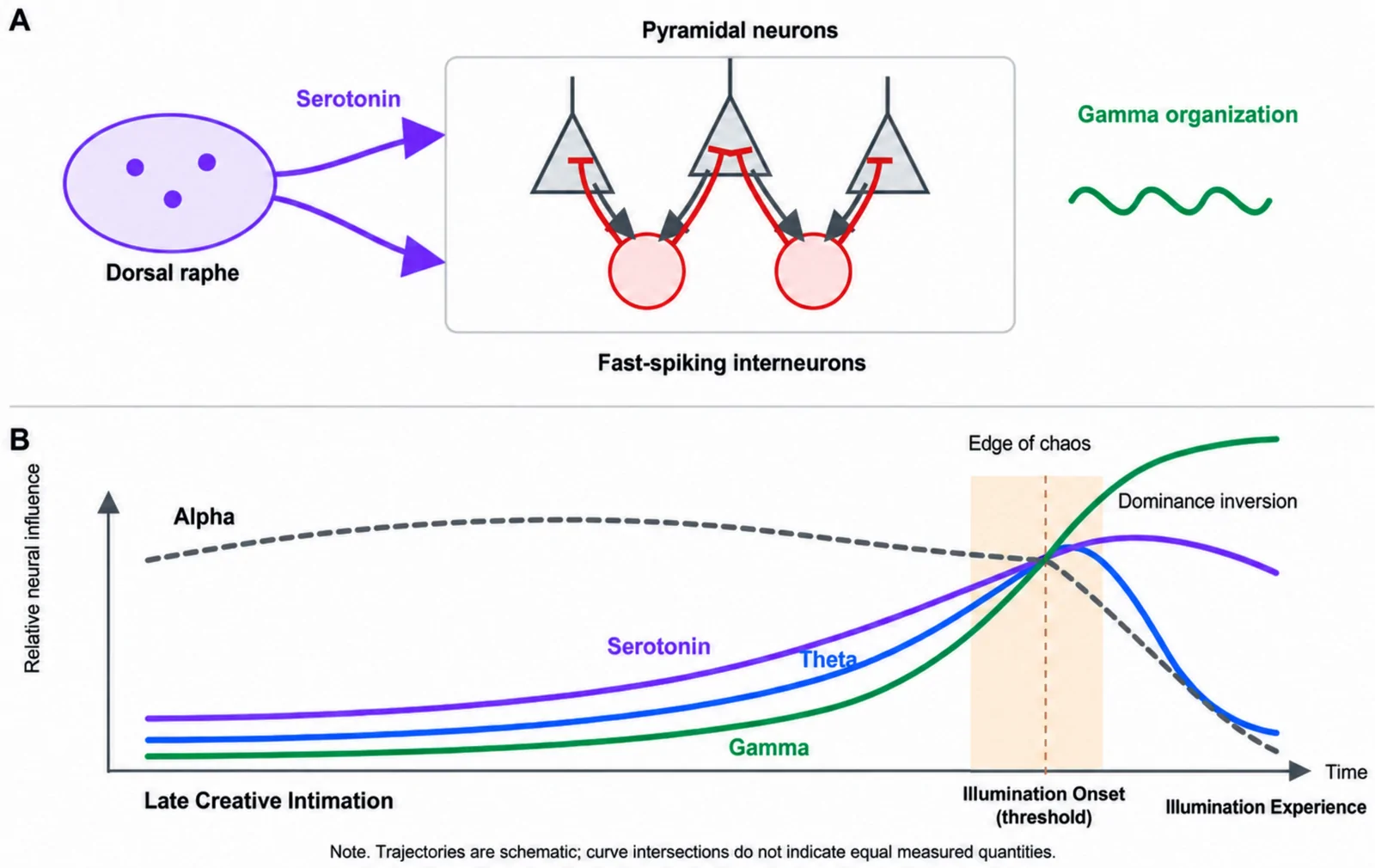
(**A**,**B**) Proposed serotonergic modulation of oscillatory organization at Illumination Onset.

**Table 1:** Stages, phases, and transitions or periods of *the Neuro-Creative Cycle*.

Stage	Phase	Transition or Period
Preparation—establishes the knowledge, goals, and problem representation that define and constrain the problem	—	—
Imagination—develops new information and relationships into a possible idea	Spontaneous Imagination	—
	Deliberate Imagination	—
	Incubation	Creative Intimation
	Illumination	Illumination Onset—*primary focus*
		Illumination Experience
Verification—tests the consciously available idea against the problem, evidence, and relevant constraints	—	—

**Table 2:** Core concepts of the illumination onset framework.

Concept	Definition
Allostasis	Anticipatory coordination of changing bodily needs across the whole person
Top-down prediction	Prior expectations guiding attention and interpretation
Bottom-up information	Embodied, emotional, imagistic, mnemonic, and associative information contributing to a new idea
Prediction error	Mismatch between a prediction and available information
Precision weighting	Relative influence assigned according to estimated reliability
Dynamic threshold	Flexible boundary at which processing changes
Edge of chaos	Vulnerable but coordinated regime near instability
Dominance inversion	The proposed shift from alpha-mediated top-down dominance to gamma-mediated bottom-up dominance at Illumination Onset

**Table 3:** Theoretical accounts integrated in the Illumination Onset framework.

Account	Established Contribution	Addition Proposed Here
Insight research (Kounios & Beeman, 2014)	Identifies activity surrounding reported insight	Distinguishes Onset from its buildup and later report
Predictive processing (Lao-Rodríguez et al., 2026)	Relates estimated reliability to the influence of evidence and expectations	Applies changing relative influence across two thresholds
Predictive routing (Gabhart et al., 2025)	Describes top-down regulation of unexpected feedforward information	Proposes that altered predictive routing contributes to the shift from alpha-mediated top-down dominance to gamma-mediated bottom-up dominance
Criticality theory (Hengen & Shew, 2025)	Characterizes marginally stable dynamics supporting information processing	Positions Onset near the edge of chaos
Neural theories of consciousness (Storm et al., 2024)	Distinguish experience, cognitive accessibility, and reporting	Identifies conscious availability of an idea as the event to be explained
Present framework	Integrates these complementary accounts	Interprets documented alpha and gamma findings as evidence relevant to the proposed dominance inversion at Illumination Onset

**Table 4:** Shared and divergent features of Illumination Onset and Seizure Onset.

Feature	Shared Characteristic	Illumination Onset	Seizure Onset
Threshold transition	Developing activity culminates in rapid reorganization	An identifiable idea becomes conscious and produces surprise	A seizure network emerges and may propagate
Cerebellar–ACC systems	Related systems are implicated in both studies	Crus I–II, ventral-DN, and ACC are proposed to support reorganization and surprise	Cerebellar output can alter seizure initiation; cingulate regions participate in some seizure networks
Oscillatory organization	Alpha–beta and gamma change around onset markers	The proposed shift from alpha-mediated top-down dominance to gamma-mediated bottom-up dominance	Inhibitory regulation becomes insufficient as pathological activity develops
Serotonin	Can alter neural responsiveness	Proposed to modulate unexpected significance and gamma coordination	Can modify seizure development
Precision weighting	Relative weighting determines which emerging activity shapes processing	Emerging information becomes dominant while earlier knowledge preserves coherence and relevance	Pathological activity can dominate processing and recruit additional regions

**Table 5:** Empirical tests of the Illumination Onset framework.

Hypothesis	Candidate Test	Supporting Result	Challenging or Refuting Result
Creative Intimation, Illumination Onset, and Illumination Experience are distinguishable, and Onset precedes its report.	Estimate Onset independently of response time using preregistered subjective, neural, behavioral, and autonomic markers and comparison trials.	Converging markers distinguish the three periods and place Onset before response initiation.	A simpler model fits better, or Onset cannot be separated from response preparation.
Intrinsic engagement and passion keep the unresolved problem meaningful after deliberate analysis relaxes.	After matched Preparation and an attention shift, test whether intrinsic engagement and passion predict maintained ratings of personal significance, problem-related thought, and later solution recognition; control knowledge, difficulty, and incentives.	Greater engagement and passion predict maintained significance, more problem-related thought, and later recognition.	None of these relationships remains after the controls are applied.
Intrinsic reward is integrated with idea recognition before later evaluation-related activity.	Separate recognition from evaluation experimentally and measure reward- and evaluation-related activity; control incentives, confidence, and success.	Reward activity coincides with recognition and precedes evaluation-related activity.	Reward occurs only later or is explained by the control variables.
Dominance inversion reflects adaptive precision weighting; theta coordinates the transition while the problem representation remains active.	Manipulate prior strength, predictability, and information reliability; use artifact-controlled EEG or magnetoencephalography (MEG) to measure oscillations, neural representations, and directed information flow; obtain independent ratings of solution fit; then perturb candidate processes.	Solution-related influence and gamma transmission increase as alpha–beta stabilization declines; theta tracks coordination, the problem representation remains detectable, and the idea satisfies the problem’s constraints.	The predicted order or information flow is absent, artifacts explain the effects, the problem representation disappears, the idea does not satisfy the problem’s constraints, or perturbation produces no predicted change.
Sensory attenuation increases the relative influence of solution-related information.	Compare predictable activity, moderately varied activity, and rest; verify attenuation, then measure the influence of solution-related and unrelated information and solution recognition while controlling cognitive demand and arousal.	Verified attenuation selectively increases solution-related influence and recognition without increasing irrelevant ideas.	Attenuation has no selective effect or its apparent effect is explained by cognitive demand or arousal.
Hemispheric contributions change across the three periods.	Compare time-resolved activity and connectivity across the periods and across verbal and nonverbal tasks.	Relatively left-weighted activity before Onset shifts toward a task-dependent right contribution near Onset and bilateral integration afterward.	No reproducible period- or task-sensitive progression occurs.
Cerebellar prediction and correction, medial-frontal attention, and ACC-mediated neural surprise form a coordinated sequence.	Combine high-spatial-resolution neuroimaging with EEG or MEG to measure model-based signals involving Crus I–II, ventral-DN, medial-frontal regions, and the ACC; then perturb candidate processes.	A mismatch persists beyond cerebellar correction and captures attention at Creative Intimation, followed by increased cerebellar–medial-frontal coordination and a stronger ACC surprise response near Onset.	The sequence is absent, does not distinguish Creative Intimation from Onset, or perturbation produces no predicted effect.
Striatal and dorsal-raphe serotonin make distinguishable contributions near Onset.	Use time-resolved neurochemical and electrophysiological measures followed by selective manipulation; control arousal and reward expectancy.	Striatal serotonin tracks intrinsic reward, whereas dorsal-raphe serotonin tracks prospective value, unexpected information, and gamma organization.	The regional or temporal distinction is absent or explained by arousal or reward expectancy.
Illumination Onset is an allostatic whole-person transition.	Synchronize neural, subjective, behavioral, and autonomic measures around Onset.	Idea recognition coincides with neural surprise and coordinated emotional and bodily changes.	The components do not coordinate reproducibly or begin only after idea recognition.
Dominance inversion occurs within a bounded near-critical range close to instability while coordination remains preserved.	Present solution-related and unrelated cues while measuring precision weighting, metastability, responsiveness, information richness, and coordination.	Selective sensitivity to solution-related information is greatest when susceptibility is high but coordination remains intact.	Recognition is unrelated to dynamical state, unrelated cues have equal effects, or sensitivity is greatest under rigid or disorganized conditions.
Precision weighting and propagation distinguish Illumination Onset from seizure onset.	Compare Illumination Onset with electrographic seizure onset, aura, clinical onset, and subsequent spread using matched measures of information content, weighting, directed influence, integration, and propagation.	Illumination preserves coordinated, information-rich processing; seizure activity escapes local regulation and spreads.	The differences disappear after controlling for movement, arousal, seizure type, and medication.

## Data Availability

The original contributions presented in this study are included in the article. Further inquiries can be directed to the corresponding author.
